# Retrospective study of influencing factors on the outcomes of luteal phase stimulation in patients with dual stimulation

**DOI:** 10.7717/peerj.15296

**Published:** 2023-05-05

**Authors:** Yuanyuan Chen, Hong Ye, Junhua Bao, Yanping Cai, Yuanbo Hu, Hongli Yan

**Affiliations:** Reproductive Medicine Center, The First Affiliated Hospital of Naval Medical University, Shanghai, China

**Keywords:** Dual stimulation (DS), Follicular phase stimulation (FPS), Luteal phase stimulation (LPS), Embryo quality

## Abstract

**Background:**

Dual/double stimulation (DS) is an ovarian stimulation strategy that has emerged in recent years; it is characterized by two rounds of ovarian stimulation and oocyte retrieval in the same menstrual cycle. DS can greatly shorten the time required to obtain valid embryos in assisted reproduction. For fertility preservation, DS can speed up oocyte storage process. However, factors influencing luteal phase ovarian stimulation (LPS) outcomes in DS have not been elucidated.

**Methods:**

A total of 156 cycles from 78 cases were studied. Patients were grouped and analyzed according to their follicular phase ovarian stimulation (FPS) types. Female ages, ovarian stimulation protocols, number of oocytes retrieved, embryo quality were recorded. Comparisons of outcomes were conducted between different groups.

**Results:**

Our study found that LPS obtained similar outcomes to follicular phase stimulation (FPS), and that the choice of FPS protocol affected the efficiency of LPS, the antagonist protocol and progestin-primed ovarian stimulation (PPOS) protocol resulted in better embryo outcomes in LPS. In LPS of DS, sufficient stimulation duration was the guarantee of embryo quality (number of available embryos: β = 0.145, 95% CI [0.078–0.211], *P* = 0.000; number of high-quality embryos: β = 0.114, 95% CI [0.057–0.171], *P* = 0.000).

**Discussion:**

This study provided ideas for the precise use of DS. We suggest to further expand the sample size of DS in the future, conduct prospective controlled studies, unify the sample size of each subgroup, include the ovarian reserve of patients in the grouping basis, and exclude the influence of male factors. We hope that this study will help further refinement of DS so as to maximize patient benefits from it.

**Conclusion:**

When the DS strategy is considered in the follicular phase, the antagonist protocol and PPOS protocol are more recommended for better embryo outcomes in LPS. During LPS, adequate ovarian stimulation duration is the most important guarantee for LPS efficiency.

## Introduction

With the understanding of multiple follicular waves within one menstrual cycle, double or triple ovarian stimulation and subsequent oocyte retrieval are being increasingly adopted by reproductive clinics (Lu et al., 2021; Polat et al., 2021; Vaiarelli et al., 2020a). Among them, dual/double stimulation (DS) is widely studied and performed, especially for women with poor ovarian responders (POR) and those in urgent need of fertility preservation. For POR with DS, patients got a better chance of getting more oocytes and subsequently more embryos for transfer (Polat et al., 2021; Sighinolfi, Sunkara & La Marca, 2018). And the cumulative live birth rate (CLBR) also doubled when compared to FPS in these patients (Vaiarelli et al., 2020a). For patients who underwent PGT-A, no significant differences were found in the euploid blastocysts rate from LPS *vs*. FPS (Ubaldi et al., 2016). For fertility preservation with DS, patients got more oocytes in less time for fertility preservation before cancer treatment (Nakasuji et al., 2019).

However, there are still no sufficient studies on how to handle DS regimens to maximize patient benefits and ensure patient safety at the same time (Tocci, 2022). So far, there is no standard process for DS and the use of DS in general population is still controversial (Lu et al., 2021).

During the past 5 years (from Jan 2017 to April 2022) in our clinics, from a total of 8,460 fresh ART cycles, 78 DS cases were screened out from ART patients regardless whether they are POR or not. In other words, for these patients, after FPS and follicular phase ovarian pick up (OPU) they have remaining follicles smaller than 12 mm and subsequently underwent secondary luteal phase ovarian stimulation for getting more oocytes and embryos within one menstrual cycle.

A retrospective case-control study was conducted for comparison of LPS outcomes among four different FPS protocols in women older than 38 years old. The numbers of oocytes retrieved and available embryos after DS were more than double those obtained after follicular-phase ovarian stimulation alone (Liu et al., 2017). However, there is still no analysis of DS outcomes in women of different ages and among different regimens undergoing assisted reproduction (Kuang et al., 2014; Vaiarelli et al., 2020b). The aim of the present study was to analyze the outcomes from the two stimulations within one menstrual cycle, including the number of oocytes retrieved and the number and rate of available and high-quality embryos. By comparing the outcomes in different women ages and among different FPS protocols, we found out two most suitable FPS protocols for maximizing DS outcomes. By using mathematical formulas, the factors influencing the outcome of LPS were given out and their weights were assessed. It is hoped that this article can provide reference for clinical DS programs.

## Materials and Methods

### Study participants and data collection

From 8,460 cycles of fresh oocyte retrieval in Reproductive Medicine Center of the First Affiliated Hospital of Naval Medical University from January 2017 to March 2022, we screened out 78 groups of DS cases within one menstrual cycle. All patients had signed an Informed Consent Form for Dual Stimulation of Ovulation Induction Cycles.

These 78 cases were grouped according to the ovarian stimulation protocols during the follicular phase. This study analyzed the outcomes of two retrievals from each of these 78 cases, comparing the number of oocytes retrieved, and the number and ratio of available and high-quality embryos among subgroups.

### Ovarian stimulation protocol

During the follicular phase, we performed a total of six ovarian stimulation options: GnRH-agonist short protocol (short protocol), GnRH-agonist long protocol (long protocol), GnRH-antagonist protocol (antagonist protocol), Progestin-primed ovarian stimulation protocol (PPOS protocol), mild-stimulation protocol and natural-cycle protocol. These protocols were performed as previously described (Schimberni et al., 2016; Xi et al., 2020; Xiao et al., 2019).

Briefly, for patients with short protocol, they received short-acting Triptorelin (Decapeptyl; Ipsen, Rome, Italy) 0.05 mg/day starting on day 1 of menstruation and subcutaneously injected with recombinant follicle stimulating hormone (rFSH) (Gonal-f; Merck Serono, Weiterstadt, Germany) or intramuscular injected with human menopausal gonadotropin (hMG) (Lizhu Pharmaceutical Factory, Zhuhai, China) 150–300 IU/day from day 2–3 of the menstruation until the hCG injection.

For patients with long protocol, they received pituitary down-regulation with a standard full dose (3.75 mg) of leuprorelin acetate microspheres (Lizhu Pharmaceutical Trading Co, Zhuhai, China) in the early follicular phase, usually day 3 of the menstruation. Patients came back 1 month later to check whether they had met the criteria for adequate suppression. The criteria for defining adequate pituitary–ovarian suppression were: E2 < 40 pg/mL, Prog < 1 ng/mL, LH < 3 IU/L, endometrial thickness <4 mm, and the absence of any growing follicle >6 mm. If the patient met the above criteria, they started controlled ovarian hyperstimulation (COH), which was performed by using rFSH or hMG at a dose of 150–300 IU/day.

Patients in the antagonist group got COH from day 2–3 of the menstruation using rFSH or hMG. When follicles with a mean diameter ≥14 mm were detected, a GnRH-antagonist (Cetrorelix; Pierre Fabre Medicament Production, Cahors, France) was injected subcutaneously at dose of 0.125–0.25 mg/day.

Patients in PPOS group were given rFSH or hMG at a dose of 150–225 IU/day and medroxyprogesterone acetate (MPA) (Zhejiang Xianju Pharmaceutical Co., Zhejiang, China) at a dose of 10 mg/day from day 3 of menstruation based on the results of the ultrasound and the blood tests.

For patients in mild-stimulation group, Clomiphene citrate (CC, Fadilan, Gaote Pharmaceuticals, Larnaca, Cyprus) was administered orally at 25–50 mg/day for 3–5 days, and some patients also injected with hMG every other day or every day until trigger day.

Follicular monitoring started 5 days later and was conducted every 2 to 3 days using a transvaginal ultrasound examination to record the number of developing follicles. Serum FSH, LH, E2, and Prog concentrations were measured using patient blood tests on the same days as the ultrasound exams. The start dose of rFSH or hMG was based on the patients age, hormone profile, AFC, body mass index (BMI) and previous treatment, and then adjusted according to E2 concentrations and ovarian responses.

For patients with natural cycle protocol, no Gn were used throughout the process, only trigger drugs were used. From day 8–10 of menstruation, transvaginal ultrasound was performed to monitor the growth of follicles.

When three dominant follicles reached 18 mm in diameter, the final stage of oocyte maturation was triggered by 0.25 mg rHCG (Ovidrel; Merck Serono, Weiterstadt, Germany) or 2000IU HCG (Lizhu Pharmaceutical Trading Co, Zhuhai, China). Transvaginal ultrasound-guided oocyte retrieval was carried out 34–36 h after the trigger.

Follicles ≤12 mm in diameter were left for the next retrieval. LPS was initiated 1–3 days after the follicular oocyte retrieval, except that GnRH-antagonist was not used to prevent LH surge.

### IVF laboratory work

According to our standard protocol, all retrieved oocytes were fertilized with either conventional IVF or ICSI. Embryos were cultured individually and scored on the third day (D3). Embryos were graded according to ASEBIR embryo assessment criteria (Alpha Scientists in Reproductive Medicine & ESHRE Special Interest Group of Embryology, 2011). All high-quality cleavage embryos were cryopreserved on D3 and the other available embryos were cultured for blastocyst until the 5^th^/6^th^ day (D5/D6). All available embryos including D3/D5/D6 from both FPS and LPS were cryopreserved with a commercial vitrification kit (Kitazato Biopharma Co., Shizuoka, Japan) on Cryotops. High-quality cleavage embryo was defined as normal fertilized embryos if they had 7–9 cleavages with less than 20% fragmentation. Available cleavage embryos on D3 were defined as normal fertilized embryos if they had five or more blastomeres and fragmentation <50%. The available blastocyst was defined as containing at most one “C” of scores for inner cell mass or trophoblast at stage 3 and above. High-quality blastocyst was defined as containing no “C” of scores for inner cell mass or trophoblast at stage 3 and above.

### Statistical analyses

The primary outcome was the number of oocytes retrieved. The secondary outcomes included the number of available and high-quality embryos, and the rate of available and high-quality embryos.

All statistical analyses were performed using IBM^®^ SPSS^®^ Statistics version 26.0 software (SPSS Inc. Headquarters, Chicago, IL, USA). Statistical comparisons were carried out using one-way ANOVA for the normally distributed data, and the Mann-Whitney U test was used for non-normally distributed data. If the variances are homogeneous, LSD method was used, and if the variances are unequal, the Tamhane method was used. For correlation studies, correlation analysis and multiple linear regression were used and the linear regression equations were given out. *P* < 0.05 was considered as statistically significant.

### Ethics

The study was approved by Reproductive Ethics Committee of the First Affiliated Hospital of Naval Medical University. The Ethical approval number is CHREC-2016-3. Informed Consent Form for Dual Stimulation of Ovulation Induction Cycles is obtained from the participants.

## Results

### Characteristics of the study participants and comparisons among subgroups

Among 8,460 fresh IVF cycles in our center, 78 cases performed DS within the same menstrual cycle. These 156 cycles counted for 1.84 percent of all our fresh cycles in the past 5 years. We did not find triple or more stimulations and oocyte retrievals within the same menstrual cycle (Gemmell, Wright & Brady, 2020). For these DS cases, the first oocyte retrieval was in follicular phase and the second was in luteal phase.

During the follicular phase, patients received different ovarian stimulation protocols, including short protocol, long protocol, antagonist protocol, PPOS protocol, mild-stimulation protocol, and natural-cycle protocol. During the luteal phase, patients received luteal phase stimulation protocol.

Table 1 showed the number of cases and distribution of various FPS protocols in each year. For different groups in FPS, nine cases performed short protocol, five cases performed long protocol, 26 cases performed antagonist protocol, 26 cases performed PPOS protocol, seven cases performed mild-stimulation protocol, and five cases performed natural-cycle protocol (Table 1).

**Table 1:** Distribution of double-cycle subgroups.

	2017	2018	2019	2020	2021	2022	Total
Short protocol	1	0	1	0	4	3	9
Long protocol	0	2	1	0	2	0	5
Antagonist protocol	1	2	6	5	8	4	26
PPOS protocol	0	8	4	6	8	0	26
Mild-stimulation protocol	0	2	3	0	2	0	7
Natural-cycle protocol	0	1	1	0	2	1	5
Total	2	15	16	11	26	8	78

**Notes:**

Number of cases and distribution of various FPS protocols in our center in the past 5 years.

DS, dual/double stimulation.

### Oocyte and embryo quality comparison between FPS and LPS in dual stimulation

All the patients in this study were for assisted reproduction. After retrieval, all the oocytes were fertilized and embryo cultured. Since the fertilization types in this study included IVF and ICSI, in terms of oocyte outcomes, we only focused on the number of oocytes retrieved, instead of the maturity of the oocytes. In addition to the number of oocytes retrieved, we paid more attention to embryo quality, including the number and the ratio of available embryos and high-quality embryos.

Previous studies reported that DS doubled the number of oocytes when compared with FPS-only and the number of oocytes retrieved in LPS was significantly higher compared to FPS, although LPS yielded a lower rate of metaphase II oocytes (Polat et al., 2021; Zhang et al., 2018). Here we further evaluated the number and the rate of available and high-quality embryos.

When comparing the outcomes of LPS with FPS, we found that the number of oocytes retrieved, the number of available embryos, and the number of high-quality embryos were slightly increased. But the rate of available embryos significantly decreased, while the rate of high-quality embryos slightly decreased in LPS (Table 2).

**Table 2:** Comparison of oocyte and embryo outcomes between FPS and LPS in DS cases.

	Mean rank			
	FPS	LPS	Z	*P*
Oocytes	76.90	80.10	−0.463	0.643
Available embryo (N)	78.48	78.52	−0.006	0.995
High quality embryo (N)	77.22	79.78	−0.401	0.689
Available embryo (rate)	85.40	71.60	−2.048	0.041*
High quality embryo (rate)	81.22	75.78	−0.842	0.400

**Notes:**

Because of the non-normal distribution of the data, the Mann-Whitney U test was used here. The number of oocytes retrieved and available embryos and high-quality embryos were slightly higher in LPS than FPS. The rate of available embryos was significantly lower in LPS than FPS. The rate of high-quality embryos was slightly lower in LPS than FPS.

* *P* < 0.05.

Oocytes: mean ranks of the number of oocytes retrieved in FPS or LPS.

Available embryos (N)/high-quality embryos (N): mean ranks of the number of available embryos and the number of high-quality embryos.

Available embryos (rate)/high-quality embryos (rate): mean ranks of the rate of available embryos and the rate of high-quality embryos.

Due to the lower rate of available embryos, we still recommend that the number of oocytes retrieving should be maximized during follicular phase to maximize the number of available embryos rather than simply rely on LPS when the patients perform DS regimen.

### Comparison of different FPS protocols on LPS outcomes of DS

Firstly, whether there was a difference in the age of the women between FPS groups was studied. Among the six FPS groups, only the women in the antagonist group were significantly younger (−4.3 years) than those in the PPOS group, which was consistent with the choice of the routine COH programs in our center: the elderly patients are more inclined to choose the PPOS protocol due to the decreased ovarian reserve (Table 3).

**Table 3:** Comparison of the basic information between different FPS groups.

	Short protocol	Long protocol	Antagonist protocol	PPOS protocol	Mild-stimulation protocol	Natural-cycle protocol	Total	F	*P*
Cases	9	5	26	26	7	5	78		
Female age (years)	39.11 ± 5.84	35.80 ± 7.12	35.62 ± 5.24^a^	39.96 ± 4.12^a^	37.00 ± 6.25	35.80 ± 5.22	37.62 ± 5.39	2.260	0.057
Retrieval interval (days)	3.56 ± 2.13^b^	2.40 ± 2.19^c^	8.12 ± 5.44^b^^,^^c^	6.15 ± 2.99	9.00 ± 4.83	11.00 ± 2.35^b^^,^^c^	6.83 ± 4.49	4.510	0.001
FPS-Gn priming dose (IU)	287.50 ± 26.52	230.00 ± 59.69	229.81 ± 47.97	258.17 ± 52.31	150.00 ± 43.30	0.00	239.38 ± 58.43	9.607	0.000
LPS-Gn priming dose (IU)	187.50 ± 45.93^d^	150.00 ± 0.00^d^	219.23 ± 47.07	258.17 ± 69.01^d^	214.29 ± 51.75	255.00 ± 41.08	225.96 ± 61.33	5.077	0.000
FPS-Gn (days)	8.78 ± 2.59	12.60 ± 2.70	7.46 ± 4.54	8.15 ± 4.13	4.43 ± 2.37	0.00	7.93 ± 4.19	3.353	0.015
LPS-Gn (days)	2.78 ± 1.79^e^	1.80 ± 1.79^e^	6.31 ± 4.52^e^	5.46 ± 3.05	6.14 ± 3.58	9.60 ± 2.51^e^	5.53 ± 3.82	3.901	0.003
FPS-Gn total dose (IU)	2,362.50 ± 885.43	3,030.00 ± 800.27	1,764.44 ± 1,311.51	2,156.25 ± 1,276.11	728.57 ± 524.49	0.00	1,965.08 ± 1,258.24	3.572	0.011
LPS-Gn total dose (IU)	568.06 ± 501.76^f^	270.00 ± 268.33^f^	1,583.65 ± 1,324.86^f^	1,483.65 ± 970.26^f^	1,323.21 ± 839.28	2,647.50 ± 819.70^f^	1,393.75 ± 1,128.87	4.039	0.003

**Notes:**

Univariate ANOVA analysis of the basic information between different FPS groups.

a Female in the antagonist group was significantly younger (−4.3) than PPOS groups.

b The interval between two oocyte retrievals in short group was significantly shorter than that in antagonist/natural-cycle group.

c The interval between two oocyte retrievals in long group was significantly shorter than that in antagonist/natural-cycle group.

d The LPS-Gn priming dose in short and long groups was significantly lower than that in PPOS group.

e The COH duration in LPS after the short and long protocol was significantly lower than that of the antagonist protocol and the natural-cycle protocol.

f The total amount of Gn used in LPS after short and long protocols was significantly lower than that in antagonist/PPOS/natural-cycle groups.

Regarding the interval between two retrievals, the interval in the short and the long protocol group was significantly shorter than that in the antagonist group and the natural-cycle group, and there was no significant difference between other groups (Table 3).

When comparing Gn priming dose in LPS, it was found that Gn priming dose in LPS after the short protocol and the long protocol was significantly lower than that in the PPOS protocol, respectively (Table 3).

When comparing the duration of COH in LPS, it was found that the COH duration in LPS after the short protocol and the long protocol was significantly lower than that of the antagonist protocol and the natural-cycle protocol (Table 3).

When comparing the total amount of Gn used in LPS, it was found that the total amount of Gn in LPS after the short protocol and the long protocol was significantly lower than that of the antagonist protocol, the PPOS protocol and the natural-cycle protocol, respectively. This was consistent with the shorter stimulation duration in these two groups (Table 3).

When comparing the number of oocytes retrieved among each group, there was no difference in either FPS or LPS (Table 4).

**Table 4:** Comparison of the FPS/LPS outcomes among different FPS group.

	Short protocol	Long protocol	Antagonist protocol	PPOS protocol	Mild-stimulation protocol	Natural-cycle protocol	Total	F	*P*
FPS-oocytes	1.11 ± 0.60	1.60 ± 1.34	1.88 ± 2.01	1.35 ± 0.75	1.71 ± 1.25	0.60 ± 0.55	1.50 ± 1.38	1.091	0.373
LPS-oocytes	0.78 ± 0.97	4.20 ± 6.61	2.50 ± 2.93	1.62 ± 1.55	2.29 ± 1.70	2.60 ± 1.14	2.10 ± 2.62	1.487	0.205
FPS-available embryo (N)	0.56 ± 0.73	0.80 ± 0.84	0.73 ± 0.92	0.81 ± 0.75	1.00 ± 1.16	0.20 ± 0.45	0.73 ± 0.83	0.670	0.648
LPS-available embryo (N)	0.11 ± 0.33^a^	1.00 ± 1.73	1.12 ± 1.61^a^	0.73 ± 0.78	1.29 ± 1.38	1.00 ± 1.23	0.87 ± 1.24	1.129	0.353
FPS-high quality embryo (N)	0.22 ± 0.44	0.60 ± 0.55	0.54 ± 0.76^b^	0.58 ± 0.70^b^	1.00 ± 1.16	0.00^b^	0.53 ± 0.73	1.490	0.204
LPS-high quality embryo (N)	0.11 ± 0.33	1.00 ± 1.73	0.88 ± 1.24	0.46 ± 0.65	1.14 ± 1.22	0.80 ± 1.30	0.68 ± 1.05	1.358	0.250
FPS-available embryo (rate, %)	44.44 ± 52.71	40.00 ± 43.53	42.35 ± 47.45	59.62 ± 47.19	52.43 ± 50.41	20.00 ± 44.72	47.67 ± 47.51	0.770	0.575
LPS-available embryo (rate, %)	11.11 ± 33.33	25.00 ± 43.30	30.73 ± 35.07	32.31 ± 37.08	37.14 ± 39.84	46.60 ± 50.58	30.22 ± 37.19	0.742	0.594
FPS-high quality embryo (rate, %)	22.22 ± 44.10	33.20 ± 40.83	30.81 ± 42.77^b^	41.00 ± 46.25^b^	52.43 ± 50.41	0.00^b^	33.33 ± 43.78	1.150	0.342
LPS-high quality embryo (rate, %)	11.11 ± 33.33	25.00 ± 43.30	26.77 ± 34.37	20.96 ± 34.02	34.29 ± 38.62	26.60 ± 43.45	23.58 ± 34.95	0.426	0.829

**Notes:**

Univariate ANOVA analysis of the FPS/LPS outcomes among different FPS group. There was no difference in the number of oocytes retrieved among each group in either FPS or LPS. There was no difference in the number of available embryos in FPS.

a In LPS, the number of available embryos in short protocol was significantly lower (−1) than that in antagonist protocol.

b The rate of available embryos did not differ between groups in the first or the second cycle. For the number and the rate of high-quality embryos, compared with the natural-cycle group, the antagonist group and the PPOS group showed higher results in follicular phase.

There was no significant difference between the groups in luteal phase.

We further compared the number of available embryos, and the results showed that there was no difference between the groups in FPS. However, in LPS, the number of available embryos in short protocol was slightly lower (−1) than that in antagonist protocol. In addition, there was also no difference in the rate of available embryos between groups in FPS or LPS (Table 4).

For the number and the rate of high-quality embryos, compared with the natural-cycle group, the antagonist group and the PPOS group showed better results in follicular phase. However, in the luteal phase there was no significant difference between the groups (Table 4).

According to the above comparisons of LPS outcomes among various FPS groups, when considering DS, antagonist or PPOS protocol was more recommended according to the patients’ condition in the first cycle. For younger patients with better ovarian reserve, antagonist protocol was the first choice. For patients with reduced ovarian reserve, PPOS protocol was recommended as FPS program. However, we did not recommend natural-cycle protocol in FPS when patient consider the second retrieval in the same menstrual cycle.

### Study on the influencing factors of LPS outcome in DS

After comparing the LPS outcomes among different FPS protocols, we also evaluated the effects of female age, LPS-Gn duration, LPS-total Gn dose, and two retrievals interval on LPS outcomes.

A total of nine DS cases in short protocol group. Only a 44-year-old patient who stimulated with Gn for 6 days (with an interval of 8 days between two retrievals) obtained one available and high-quality embryo in the second cycle. This patient was also the one who received the longest LPS in this group.

A total of five DS cases in long protocol group, and two obtained available and high-quality embryos in luteal phase. One was a 28-year-old woman, who got 16 oocytes by LPS, resulting in four available and high-quality embryos. Another 44-year-old woman got one available and high-quality embryo, and this patient also received the longest stimulation (5 days) in this group.

Similar conclusions were found in antagonist protocol, PPOS protocol, mild-stimulation protocol, and natural-cycle protocol groups. The number of oocytes retrieved after LPS was negatively correlated with the woman’s age, and positively correlated with the duration and the total amount of Gn in LPS. The number and the rate of available and high-quality embryos was positively correlated with the interval between two retrievals, the duration and the total amount of Gn in LPS, and the strongest correlation was the duration of LPS (Table 5).

**Table 5:** Correlation between influencing factors and outcomes of LPS in DS.

Correlations						
		Oocytes (N)	Available embryo (N)	High quality embryo (N)	Available embryo (rate)	High quality embryo (rate)
Age	Pearson correlation	−0.289*	−0.142	−0.158	0	0.017
	Sig. (2-tailed)	0.01	0.217	0.168	0.998	0.883
	N	78	78	78	78	78
Interval	Pearson correlation	0.18	0.386**	0.363**	0.424**	0.355**
	Sig. (2-tailed)	0.114	0	0.001	0	0.001
	N	78	78	78	78	78
LPS-Gn priming dose	Pearson correlation	0.09	0.168	0.141	0.225*	0.189
	Sig. (2-tailed)	0.432	0.142	0.219	0.047	0.098
	N	78	78	78	78	78
LPS-Gn days	Pearson correlation	0.224*	0.445**	0.415**	0.432**	0.348**
	Sig. (2-tailed)	0.048	0	0	0	0.002
	N	78	78	78	78	78
LPS-Gn total dose	Pearson correlation	0.264*	0.421**	0.379**	0.412**	0.327**
	Sig. (2-tailed)	0.02	0	0.001	0	0.004
	N	78	78	78	78	78

**Notes:**

* Correlation is significant at the 0.05 level (2-tailed).

** Correlation is significant at the 0.01 level (2-tailed).

Next, linear regression on LPS outcomes and influencing factors was performed. Firstly, regardless of the FPS protocols, the outcomes were regressed on the age of the woman, the interval between two retrievals, the duration of stimulation and the total amount of Gn (Table 6).

**Table 6:** Linear regression on LPS outcomes and influencing factors regardless of the FPS protocols.

		Model		Coefficients									
		Adjusted R square	F	Sig.	Unstandardized coefficients		Standardized coefficients	t	Sig.	95% confidence interval for B		Collinearity statistics	
Model					B	Std. error	Beta			Lower bound	Upper bound	Tolerance	VIF
Oocytes	(Constant)	0.130	6.754	0.002	6.525	1.997		3.267	0.002	2.546	10.503		
	Female age (age)				−0.140	0.052	−0.288	−2.709	0.008*	−0.243	−0.037	1	1
	LPS-Gn dose (Gn)				0.001	0.000	0.263	2.475	0.016*	0.000	0.001	1	1
Available embryos (LPS-AE)	(Constant)	0.187	18.773	0.000	0.072	0.224		0.323	0.748	−0.374	0.518		
	LPS-Gn days (GnD)				0.145	0.033	0.445	4.328	0.000*	0.078	0.211	1	1
High-quality embryos (LPS-HQE)	(Constant)	0.161	15.819	0.000	0.048	0.193		0.250	0.803	−0.335	0.432		
	LPS-Gn days (GnD)				0.114	0.029	0.415	3.977	0.000*	0.057	0.171	1	1

**Note:**

* *P* < 0.05.

The regression equation for the number of oocytes retrieved and the female age (years old) and total Gn dose (IU) in LPS is:

$\rm{Oocytes = 6.525 - 0.14*age + 0.001*Gn}$

The regression equations for the number of available and high-quality embryos (LPS-AE/LPS-HQE) and the duration of Gn administration (GnD) in LPS were:

$\rm{LPS{-}AE = 0.072 + 0.145*GnD}$

$\rm{LPS{-}HQE = 0.048 + 0.114*GnD}$

When including FPS regimens, linear regression on LPS oocytes retrieved and influencing factors showed that the long protocol had significant protective effects compared with the antagonist protocol, the total amount of LPS-Gn was a protective factor while the age of the woman was a risk factor. As to the number of available and high-quality embryos, Gn duration of LPS was the only protective factor, and the effect of FPS protocol selection was not obvious (Table 7).

**Table 7:** Linear regression on LPS outcomes and influencing factors including FPS regimens.

		Model			Coefficients								
		Adjusted R square			Unstandardized coefficients		Standardized coefficients			95% confidence interval for B		Collinearity statistics	
Model			F	Sig.	B	Std. error	Beta	t	Sig.	Lower bound	Upper bound	Tolerance	VIF
Oocytes	(Constant)	0.153	2.993	0.008	5.278	2.268		2.327	0.023	0.755	9.802		
	age				−0.121	0.055	−0.248	−2.197	0.031*	−0.230	−0.011	0.863	1.159
	LPS-Gn				0.001	0.000	0.337	2.842	0.006*	0.000	0.001	0.780	1.282
	protocol = 1.0				−0.223	0.967	−0.027	−0.230	0.818	−2.152	1.706	0.781	1.280
	protocol = 2.0				3.033	1.243	0.285	2.440	0.017*	0.553	5.513	0.804	1.243
	protocol = 4.0				0.281	0.711	0.051	0.396	0.694	−1.137	1.700	0.664	1.507
	protocol = 5.0				0.438	1.041	0.048	0.421	0.675	−1.637	2.514	0.843	1.186
	protocol = 6.0				−0.430	1.244	−0.040	−0.345	0.731	−2.912	2.052	0.803	1.245
Available embryos (LPS-AE)	(Constant)	0.187	3.942	0.002	0.141	0.324		0.437	0.663	−0.504	0.787		
	LPS-GnD				0.154	0.038	0.475	4.095	0.000*	0.079	0.230	0.787	1.271
	protocol = 1.0				−0.459	0.453	−0.119	−1.014	0.314	−1.363	0.444	0.767	1.303
	protocol = 2.0				0.581	0.573	0.115	1.014	0.314	−0.561	1.723	0.817	1.224
	protocol = 4.0				−0.254	0.312	−0.097	−0.813	0.419	−0.877	0.369	0.742	1.347
	protocol = 5.0				0.196	0.477	0.045	0.411	0.683	−0.755	1.147	0.865	1.156
	protocol = 6.0				−0.624	0.561	−0.124	−1.112	0.270	−1.742	0.494	0.852	1.173
High-quality embryos (LPS-HQE)	(Constant)	0.187	3.959	0.002	0.141	0.324		0.437	0.663	−0.504	0.787		
	LPS-GnD				0.154	0.038	0.475	4.095	0.000*	0.079	0.230	0.787	1.271
	protocol = 1.0				−0.459	0.453	−0.119	−1.014	0.314	−1.363	0.444	0.767	1.303
	protocol = 2.0				0.581	0.573	0.115	1.014	0.314	−0.561	1.723	0.817	1.224
	protocol = 4.0				−0.254	0.312	−0.097	−0.813	0.419	−0.877	0.369	0.742	1.347
	protocol = 5.0				0.196	0.477	0.045	0.411	0.683	−0.755	1.147	0.865	1.156
	protocol = 6.0				−0.624	0.561	−0.124	−1.112	0.270	−1.742	0.494	0.852	1.173

**Notes:**

Protocol = 1.0: short protocol, protocol = 2.0: long protocol, protocol = 3.0: antagonist protocol, protocol = 4.0: PPOS protocol, protocol = 5.0: mild-stimulation protocol, protocol = 6.0: natural-cycle protocol.

* *P* < 0.05.

The above regression analysis showed that for LPS in DS, sufficient duration of stimulation should be ensured to maximize available and high-quality embryo acquisition.

### Analysis of DS pregnancy outcomes

Due to the limited sample size, we preliminarily analyzed pregnancy outcomes in the samples of this study. There were no successful pregnancy cases in the groups of short, long, and natural-cycle protocols. In the mild-stimulation group, FPS and LPS each obtained one clinical pregnancy, but early spontaneous abortion happened in the case of LPS. In the antagonist group, there were nine transplants in FPS and LPS respectively, including four and two live births, two and four cases of early spontaneous abortion, and three cases of non-pregnancy respectively. In the PPOS group, there were 12 and seven transplants in FPS and LPS respectively, including four and one live birth, one case of early spontaneous abortion respectively, and seven and five cases of non-pregnancy (Table 8). Overall, similar to embryonic outcomes, pregnancy outcomes of FPS were better than LPS in DS. However, LPS can still lead to successful live births, and the success cases were all from antagonist and PPOS groups. Therefore, based on the preliminary comparisons of clinical live birth outcomes, we still recommend the use of antagonist and PPOS protocols for DS.

**Table 8:** Pregnancy outcomes of DS.

Cases		Total	Live birth	Early abortion	Non-pregnancy
Antagonist group	FPS	9	4	2	3
	LPS	9	2	4	3
PPOS group	FPS	12	4	1	7
	LPS	7	1	1	5

## Discussion

Previous Meta-analysis demonstrated that LPS could achieve pregnancy outcomes non-inferior to FPS group, despite that LPS required a longer duration of stimulation and a higher dose of Gn (Boots et al., 2016; Lu et al., 2021). In this study, we conducted an in-depth group exploration on dual stimulation. First, we compared the oocyte retrieval and embryo outcomes of the two cycles in DS. LPS yielded slightly more oocytes, available embryos, and high-quality embryos than FPS, but the available embryo rate and the high-quality embryo rate were slightly lower. Overall, DS can significantly improve patient benefits from a single cycle, doubling the patient’s available embryos.

Then, we divided DS into different groups according to FPS protocols and explored the effects of different FPS protocols on LPS outcomes. We found that the antagonist and PPOS groups yielded better outcomes in LPS, and although patients in antagonist group were significantly younger than PPOS group, there was no significant difference in LPS outcomes between these two groups. For patients who choose DS, we recommend antagonist or PPOS protocols in follicular phase according to their own conditions.

In addition to discussing LPS outcomes among different FPS groups, we further studied the effects of age and stimulation characteristics on LPS outcomes. Although in terms of the number of oocytes retrieved, youngers had an obvious advantage, that is, the younger the woman is, the more oocytes retrieved by LPS, and vice versa. But in the indicators of available and high-quality embryos, age did not affect the outcomes. The stimulus duration, the total amount of Gn in LPS and the interval between two retrievals are all significantly correlated to the embryo outcomes, among which the stimulus duration has the strongest correlation. Therefore, we recommend sufficient full-stimulation in LPS of the DS to maximize the available and high-quality embryos.

In this study we selected DS cases in our center from the past 5 years. Since the DS program has emerged in recent years and due to the limited sample size, we preliminarily analyzed the pregnancy outcomes without statistical analysis. Encouragingly, they were consistent with embryonic outcomes. We will further refine the study by including influencing factors such as women’s ovarian reserve, male’s fertility and types of fertilization to get more solid conclusions.

It is believed that compared with the traditional program, DS program can indeed increase the benefits for patients in short term and improve the efficiency of assisted reproduction. With the development of precision medicine, we should further refine the treatment of patients, choose the most suitable programs, and improve the efficiency of assisted reproduction, so as to achieve the goal of having a healthy baby as soon as possible.

On the other hand, with the development of oncology, more and more patients need fertility preservation before tumor treatment (Vaiarelli et al., 2020b). Choosing an efficient fertility preservation method is bound to have significant impacts on tumor treatment and future fertility. DS might be considered as an extremely promising method for women with cancer when they consider oocyte retrieval for fertility preservation. We will further explore and optimize DS to provide the most effective protocol for fertility preservation, so as to obtain as many oocytes and embryos as possible in the shortest time.

## Supplemental Information

10.7717/peerj.15296/supp-1 Supplemental Information 1 Individual raw measurements.

10.7717/peerj.15296/supp-2 Supplemental Information 2 Raw data.

## Additional Information and Declarations

### Data Availability

The following information was supplied regarding data availability: The data is available at Figshare: Chen, Yuanyuan; Hong, Ye; Bao, Junhua; Cai, Yanping; Hu, Yuanbo; Yan, Hongli (2022): Analysis of influencing factors on the outcomes of luteal phase stimulation in patients with dual stimulation-tables.docx. figshare. Dataset. https://doi.org/10.6084/m9.figshare.21366201.v1.
